# Tuberculosis in a Cow

**Published:** 1882-07

**Authors:** Thomas A. Allen


### VI.—TUBERCULOSIS IN A COW.

REPORTED BY THOMAS A. ALLEN, M.D.

In the Spring of 1880 I was called to see a common bred cow that had been troubled with a cough and gradual emaciation for a year. When I arrived, she was lying on her right side, breathing very rapidly. The emaciation and weakness had reached such a degree that she was unable even to raise her head from the ground.

Convinced that there was no chance of recovery, I severed the jugulars and allowed her to bleed to death. She bled freely, and the blood was very dark in color.

*Necropsy* immediately after death.—Right lung a complete mass of tuberculous matter or nodules. The left lung was in nearly the same condition, excepting one portion, about the size of a man's head, which remained normal.

The *diaphragm* was thickened by these tuberculous nodules, and measured eighteen centimetres in thickness. The *costal* pleuræ were thickly studded with the same kind of nodules. The *peritoneum*, both visceral and parietal layers, were thickly studded with tubercular masses, varying in size from a millet seed to a large cucumber.

The whole mass of new development would weigh about thirty pounds.

BROCKVILLE, ONT., CANADA, April 28th, 1882.

